# Percutaneous transluminal renal angioplasty with stenting for stenotic venous bypass grafts: report of two cases

**DOI:** 10.1186/2193-1801-2-456

**Published:** 2013-09-12

**Authors:** Masashi Kusakabe, Hiroki Sasaki, Jiro Sato, Masaaki Akahane, Tetsuro Miyata, Kuni Ohtomo

**Affiliations:** Department of Radiology, Graduate School of Medicine, The University of Tokyo, 7-3-1, Hongo, Bunkyo-ku, Tokyo, Japan; Division of Vascular Surgery, Department of Surgery, Graduate School of Medicine, The University of Tokyo, 7-3-1, Hongo, Bunkyo-ku, Tokyo, Japan

**Keywords:** Angioplasty, Renal artery, Stents, Venous grafts

## Abstract

Cases of percutaneous transluminal renal angioplasty for renal artery stenosis are increasing. However, percutaneous transluminal renal angioplasty with stenting for stenotic venous bypass grafts has never been reported. Herein, the authors describe two cases of percutaneous transluminal renal angioplasty with stenting for a stenotic venous bypass graft. The patients in both cases had undergone bypass grafting using autologous saphenous veins, which were anastomosed directly to their abdominal aortas. We successfully conducted percutaneous transluminal renal angioplasty with stenting. One of the keys for technical success is an appropriate selection of guiding catheter compatible with postoperative nonanatomical vasculature, and the other is relatively high pressure dilation for venous stenosis.

## Background

Percutaneous transluminal renal angioplasty (PTRA) was first described in 1978 (Gruntzig et al. 1978), and has been conducted for the treatment of renal artery stenosis for more than thirty years. Cases of PTRA for renal artery stenosis are increasing with the increase of atherosclerotic diseases and recognition of renovascular hypertension.

Other than PTRA, surgical procedures are also performed for renal vascular diseases and have the same problem, restenosis of treated vessels, as PTRA. In the field of coronary artery, percutaneous coronary intervention (PCI) for stenotic venous bypass grafts is generally conducted as one of the way of salvage (Hernandez-Antolin et al. 2009; Hong et al. 2001; De Feyter et al. 1993). There is a report about PTRA for stenotic venous bypass grafts (Garfinkel et al. 1984). However, the detailed procedure was not mentioned in the literature and PTRA with stenting for stenotic venous bypass grafts has never been reported.

We successfully conducted PTRA with stenting for stenotic venous bypass grafts in two cases. Herein, we describe those two cases and review the keys for technical success.

## Case reports

### Case one

**Figure 1:**
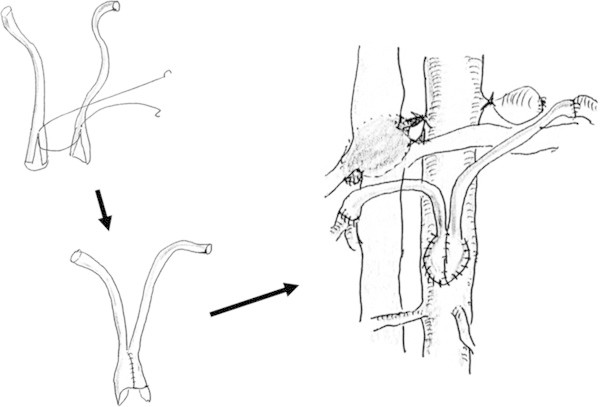
**Scheme of bilateral renal artery bypass grafting using autologous saphenous vein grafts.**

Institutional review board approved case reports at our institution. A 64-year-old woman was referred to our department for PTRA. She underwent patch plasty of bilateral renal arteries using autologous saphenous veins for fibromuscular dysplasia in her twenties. Around 30 years after the operation, bilateral renal artery aneurysms were detected. Bilateral renal artery bypass grafting was performed using autologous saphenous vein grafts (Figure 1). Two harvested saphenous veins were anastomosed at each end to be shaped as pouch, and the pouch was anastomosed directly to her abdominal aorta distal to the renal artery branches. Two years after the bypass operation, follow-up renal ultrasonography revealed significant stenosis at the left renal bypass graft (peak systolic velocity: 474.2 cm/sec, renal aortic ratio: 5.5, and resistive index: 0.47-0.63), and renal perfusion scintigraphy revealed that left renal perfusion was decreased. Before PTRA, blood pressure was 148/86 with three types of antihypertensive drugs (Amlodipine Besylate 5 mg/day, Candesartan Cilexetil 8 mg/day, Atenolol 25 mg/day). Creatinine clearance was 81.1 ml/min, and resistive index of the left renal artery was mentioned above. Therefore, her renal function was thought to be maintained, and this case was indicated for PTRA.

**Figure 2:**
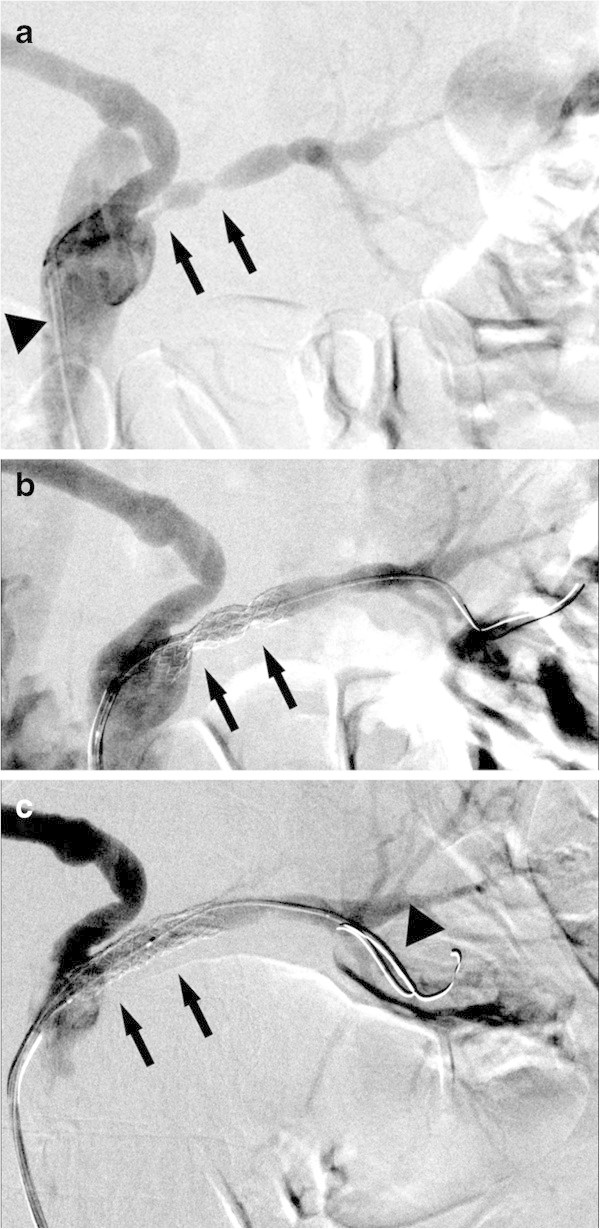
**PTRA for the left stenotic venous bypass graft. a**. Angiography before PTRA. We used a guiding catheter which shape of the tip was relatively straight (arrowhead). There were two sites of stenosis at the left renal bypass graft (arrow). **b**. Angiography after stenting. The mounted balloon could not achieve adequate dilation (arrow). **c**. Angiography after additional dilation. Stenotic lesions were adequately dilated (arrow). We used double wire technique to insert the additional dilator (arrowhead).

We started PTRA with left femoral approach and placed a 6-F long sheath. To keep the root of the reconstructed venous graft, we used a 6-F guiding catheter (ENVOY, Cordis, Bridgewater, NJ, USA) which shape of the tip was relatively straight (Multipurpose D). Two sites of stenosis were detected at the left renal bypass graft (Figure 2a). We passed a 0.014-inch microguidewire (Cruise, St. Jude Medical, St. Paul, MN, USA) through the stenotic region, and performed a predilation at 8 atm using a 2.6-F balloon (3 mm × 40 mm, IKAZUCHI PAD, KANEKA MEDIX, Osaka, Japan). After the predilation, two stents were deployed with the mounted balloons (Amiia, Cordis, Bridgewater, NJ, USA) at 12 atm respectively. One stent (6 mm × 15 mm, PALMAZ Genesis, Cordis, Bridgewater, NJ, USA) was on the distal stenosis and the other stent (6 mm × 18 mm, PALMAZ Genesis, Cordis, Bridgewater, NJ, USA) was on the proximal stenosis. The mounted balloons were 6-F balloons with a rated burst pressure of 12 atm. Because residual stenoses were significant despite those stents were deployed at the rated burst pressure of the mounted balloon (Figure 2b), another slightly higher pressure balloon with a rated burst pressure of 14 atm (6 mm × 20 mm, Aviator Plus, Cordis, Bridgewater, NJ, USA) was inserted using double wire technique (Chetcuti and Moscucci 2004) and additional dilations twice at 14 atm with the Aviator Plus achieved adequate dilation (Figure 2c). We used double wire technique because we could not insert the balloon with a single wire. Two years after the PTRA, blood pressure is 128/72 with antihypertensive drugs (Amlodipine Besylate 2.5 mg/day, Candesartan Cilexetil 4 mg/day). Creatinine clearance is 69.3 ml/min, and follow-up computed tomography reveals no restenosis.

### Case two

**Figure 3:**
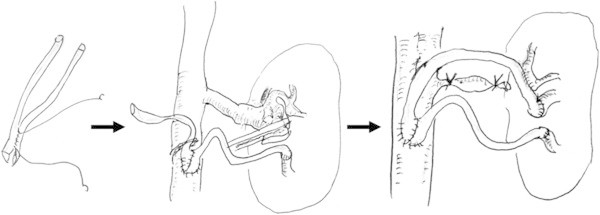
**Scheme of left renal artery bypass grafting using autologous saphenous vein grafts.** Each peripheral end of the saphenous veins was anastomosed to anterior and posterior branch of the left renal artery respectively.

The second case is very similar with the first case mentioned above. A 59-year-old man was referred to our department for PTRA. He underwent right nephrectomy and patch plasty of left renal artery using an autologous saphenous vein for fibromuscular dysplasia and induced right renal complete dysfunction in his twenties. Around 30 years after the operation, a left renal artery aneurysm was detected. Left renal artery bypass grafting was performed using autologous saphenous vein grafts (Figure 3). Two harvested saphenous veins were anastomosed at each end to be shaped as pouch, and the pouch was anastomosed directly to his abdominal aorta distal to the left renal artery branch. Three years after the bypass operation, follow-up renal ultrasonography revealed significant stenosis at the posterior branch of the left renal bypass graft (peak systolic velocity: 527.3 cm/sec, renal aortic ratio: 6.2, and resistive index: 0.38-0.63), and PTRA was planned. Blood pressure was 130/76 before the PTRA with two types of antihypertensive drugs (Amlodipine Besylate 2.5 mg/day, Losartan potassium 50 mg/day). Creatinine clearance was 60.6 ml/min and his renal function was thought to be maintained.

**Figure 4:**
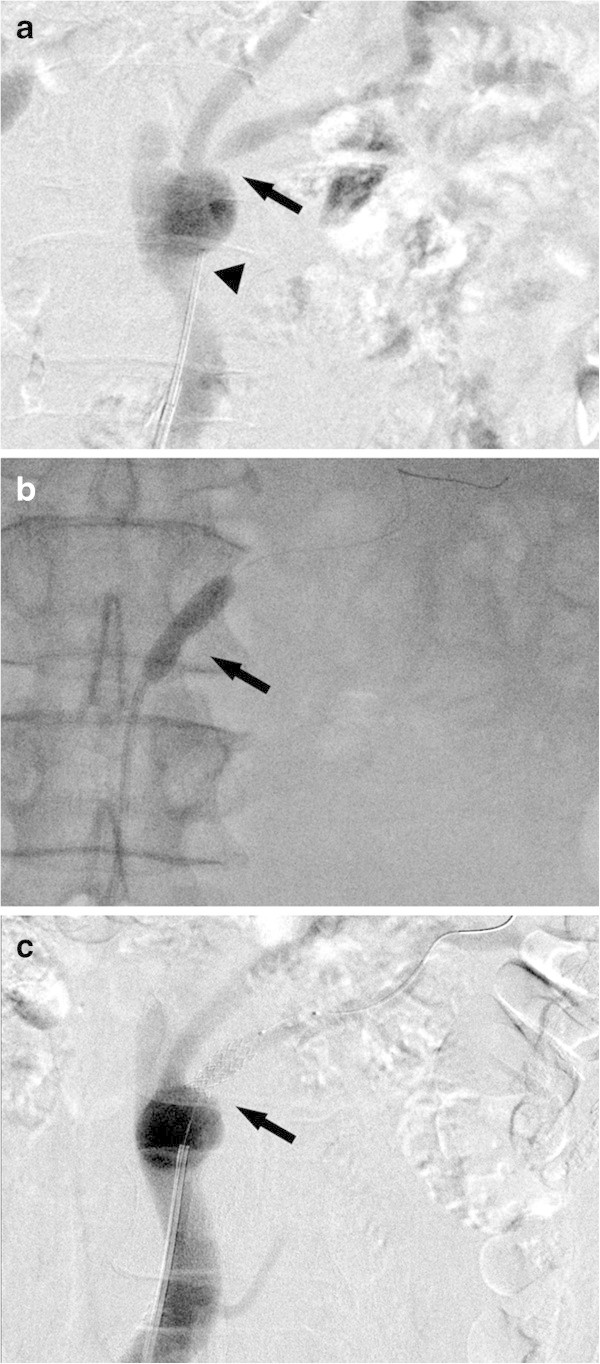
**PTRA for the stenotic posterior branch of the venous bypass graft. a**. Angiography before PTRA. We used carbon dioxide gas as contrast material because the creatinine level of the patient was elevated to 1.27 mg/dl. We used a guiding catheter which shape of the tip was straight (arrowhead). There was stenosis at the proximal region of the posterior branch of the left renal bypass graft (arrow). The anterior branch of the left renal bypass graft showed no significant stenosis. **b**. The mounted balloon could not achieve adequate dilation (arrow). **c**. Angiography after additional dilation. Stenotic lesion was adequately dilated (arrow).

We started PTRA with left femoral approach and placed a 6-F long sheath. To keep the root of the reconstructed venous graft, we used a 6-F guiding catheter (Mach1, Boston Scientific, Natick, MA, USA) which shape of the tip was straight. Stenosis was detected at the proximal region of the posterior branch of the left renal bypass graft (Figure 4a). We passed a 0.014-inch microguidewire (Agosal XS 0.8, St. Jude Medical, St. Paul, MN, USA) through the stenotic region, and performed a predilation at 8 atm using a balloon (4 mm × 20 mm, Aviator Plus, Cordis, Bridgewater, NJ, USA). After the predilation, a stent (6 mm × 16 mm, PALMAZ Genesis, Cordis, Bridgewater, NJ, USA) was deployed with the mounted balloon (Amiia, Cordis, Bridgewater, NJ, USA) at 12 atm. Because the mounted balloon could not achieve adequate dilation (Figure 4b), another balloon (6 mm × 15 mm, Aviator Plus, Cordis, Bridgewater, NJ, USA) was inserted and additional dilation at 14 atm achieved adequate dilation (Figure 4c). Two months after the PTRA, blood pressure is 126/66 and antihypertensive drugs which have been taken for 11 years can be ceased. Creatinine clearance is 61.5 ml/min, and follow-up renal ultrasonography reveals no restenosis.

## Discussion

Although there are many reports about PCI for stenotic venous bypass grafts (Hernandez-Antolin et al. 2009; Hong et al. 2001; De Feyter et al. 1993), there is only one report about PTRA for stenotic venous bypass grafts (Garfinkel et al. 1984). However, the detailed procedure was not mentioned in the literature and PTRA with stenting for stenotic venous bypass grafts has never been reported. Considering that cases of atherosclerotic disease are increasing, cases of venous bypass grafting for atherosclerotic renal artery aneurysm and PTRA for stenotic venous bypass grafts will increase in the future.

In each of our two cases, PTRA was performed for the stenotic venous bypass graft, which had been grafted for the treatment of renal artery aneurysm and was anastomosed directly to the abdominal aorta distal to the renal artery branches nonanatomically (Figures 1 and 3). Although we generally use a guiding catheter which shape of the tip is curved such as renal double curve or hocky stick type in PTRA (Zeller and Schwarzwalder 2009), we selected a guiding catheter which shape of the tip is relatively straight compatible with nonanatomical vasculature and could keep the root of the reconstructed venous graft successfully (Figures 2a and 4a). We used the different type of guiding catheter in two cases. It is because the shape of the proximal side of anastomosed venous bypass graft was different in two cases and we selected the appropriate guiding catheter compatible with postoperative nonanatomical vasculature.

Compared with atherosclerotic arterial stenosis, venous stenosis requires high pressure dilation (Trerotola et al. 2005). In each of our two cases, relatively high pressure dilation (14 atm) was additionally required for adequate dilation (Figures 2c and 4c). However, excessive high pressure dilation may lead to the rupture of the vessel. Unlike superficial veins, venous bypass grafts for renal arteries are located deeply in bodies and the rupture of the vessel will need an emergent operation. We think that it is important to select the appropriate size of the balloon for the targeted vessel and to limit the pressure slightly higher than the rated burst pressure of the mounted balloon of the stent product for safe procedures.

Our report of PTRA for stenotic venous bypass grafts represents just two cases and follow-up period after PTRA is short. Therefore, long-term prognosis such as risk of restenosis or course of renal function is not clear. Protection from distal embolism is discussed in the field of PCI for stenotic venous bypass grafts, but was not preformed in our procedures. We should consider long-term follow-up and protection from distal embolism in the future.

Our findings indicate that an appropriate selection of guiding catheter compatible with postoperative nonanatomical vasculature and relatively high pressure dilation for venous stenosis are the keys for technical success in PTRA with stenting for stenotic venous bypass grafts.

## Consent

Written informed consent was obtained from the patient for the publication of this report and any accompanying images.

## Abbreviations

PTRA: Percutaneous transluminal renal angioplasty
PCI: Percutaneous coronary intervention.
